# The Effect of High Dose Isoflavone Supplementation on Serum Reverse T_3_ in Euthyroid Men With Type 2 Diabetes and Post-menopausal Women

**DOI:** 10.3389/fendo.2018.00698

**Published:** 2018-11-22

**Authors:** Thozhukat Sathyapalan, Josef Köhrle, Eddy Rijntjes, Alan S. Rigby, Soha R. Dargham, Eric S. Kilpatrick, Stephen L. Atkin

**Affiliations:** ^1^Academic Diabetes, Endocrinology and Metabolism, Hull York Medical School, University of Hull, Hull, United Kingdom; ^2^Institut für Experimentelle Endokrinologie, Charité-Universitätsmedizin Berlin, Berlin Institute of Health, CVK, Humboldt-Universität zu Berlin, Berlin, Germany; ^3^Weill Cornell Medical College Qatar, Doha, Qatar; ^4^Department of Clinical Chemistry, Sidra Medical and Research Center, Doha, Qatar

**Keywords:** soy, isoflavones, phytoestrogens, reverse tri-iodothyronine, tri-iodothyronine, thyroxine, TSH

## Abstract

**Background:** The health benefits of soy are widely reported but there are queries on the effect of soy isoflavones on thyroid function and the underlying mechanism of action.

**Materials and Methods:** We examined the effect of soy isoflavones on reverse tri-iodothyronine (or 3,3′,5′-tri-iodothyronine; rT_3_) in two studies comprising 400 patients: 200 men (study 1; 3 months) and 200 post-menopausal women (study 2; 6 months) who were randomized to consume 15 g soy protein with 66 mg of isoflavones (SPI) daily, or 15 g soy protein alone without isoflavones (SP) daily.

**Results:** SPI supplementation increased rT_3_ serum concentration in both men 0.41 (0.12) vs. 0.45 (0.14) nmol/L and women 0.33 (0.12) vs. 0.37 (0.09) nmol/L at 3 months compared to SP that was not seen at 6 months. Thyroid stimulating hormone (TSH) serum concentrations increased while free thyroxine (fT_4_) concentrations decreased with 3 months of SPI compared to SP supplementation for both men and women. rT_3_ correlated with TSH in both studies (*p* = 0.03) but not with either fT_3_ or fT_4_. fT_3_ levels did not differ between the SPI and SP preparations.

**Conclusion:** Soy isoflavones transiently increased rT3 levels within 3 months though reverted to baseline at 6 months. The mechanism for this would be either rT3 degrading deiodinase 1 and/or deiodinase 2 activities are transiently inhibited at 3 months, or inhibition of deiodinase 3, which generates rT3 from T4 is induced at 6 months. These changes were mirrored in the TSH concentrations, suggesting that short-term high dose isoflavone transiently impairs thyroid function in the first 3 months and may impact on general health during this period.

**ISRCTN Registry:** ISRCTN 90604927; ISRCTN34051237.

## Introduction

The consumption of soy food products have increased due to the reported potential health benefits that have been suggested to be due to the isoflavone components, leading to the development of isoflavone supplements and the fortification of foods with isoflavones (1, 2). It has been suggested that the soy isoflavones might provide protection against breast and prostate cancer (3–5), osteoporosis (6), cardiovascular diseases (7, 8) and alleviate hot flashes (9). However, there are concerns in susceptible individuals that soy may adversely affect thyroid function (10–14). The mechanism by which soy isoflavones may interfere with thyroid function is unclear, but it is critical to understand given the wide spread use of soy products. Animal studies have suggested that soy isoflavones interfere with thyroid function via thyroid peroxidase inhibition, as well as, with tissue deiodinase enzyme activities, which may affect extrathyroidal thyroid hormone metabolism, including rT_3_ concentrations (15). Isoflavones might also displace thyroid hormones from their distributor proteins in the blood (16).

We have shown that high dose isoflavone intake, in comparison with isoflavone free soy, impair thyroid function in patients with type 2 diabetes (T2DM) (Study 1) (17) and also in post-menopausal healthy women (Study 2) (18). Study 1 was a randomized double-blind parallel study investigating the effect of soy isoflavones on testosterone serum concentrations in men with T2DM; 3 months of high dose isoflavone intake resulted in a significant increase in serum concentrations of thyrotropin (TSH) and a reduction of free thyroxine (fT_4_) with no changes in serum free tri-iodothyronine (fT_3_) concentration. Similarly, in study 2, a double-blind randomized parallel study investigating the effect of high dose isoflavone intake on bone turnover markers in women within 2 years of onset of menopause, 6 months of high dose isoflavone resulted in a significant increase in TSH and reduction of fT_4_ with no changes in fT_3_. As rT_3_ is a major endogenous T_4_ metabolite, probably devoid of major biological action in adults, we analyzed potential rT_3_ concentration changes in serum, which might be harbingers of altered thyroid hormone distribution and metabolism (19). We conducted this *post-hoc* analysis to understand the impact of high dose isoflavones on rT_3_ concentrations and their correlation with other thyroid measurement parameters tests including fT_3_, fT_4_ and TSH.

## Research design and methods

**Figure 1:**
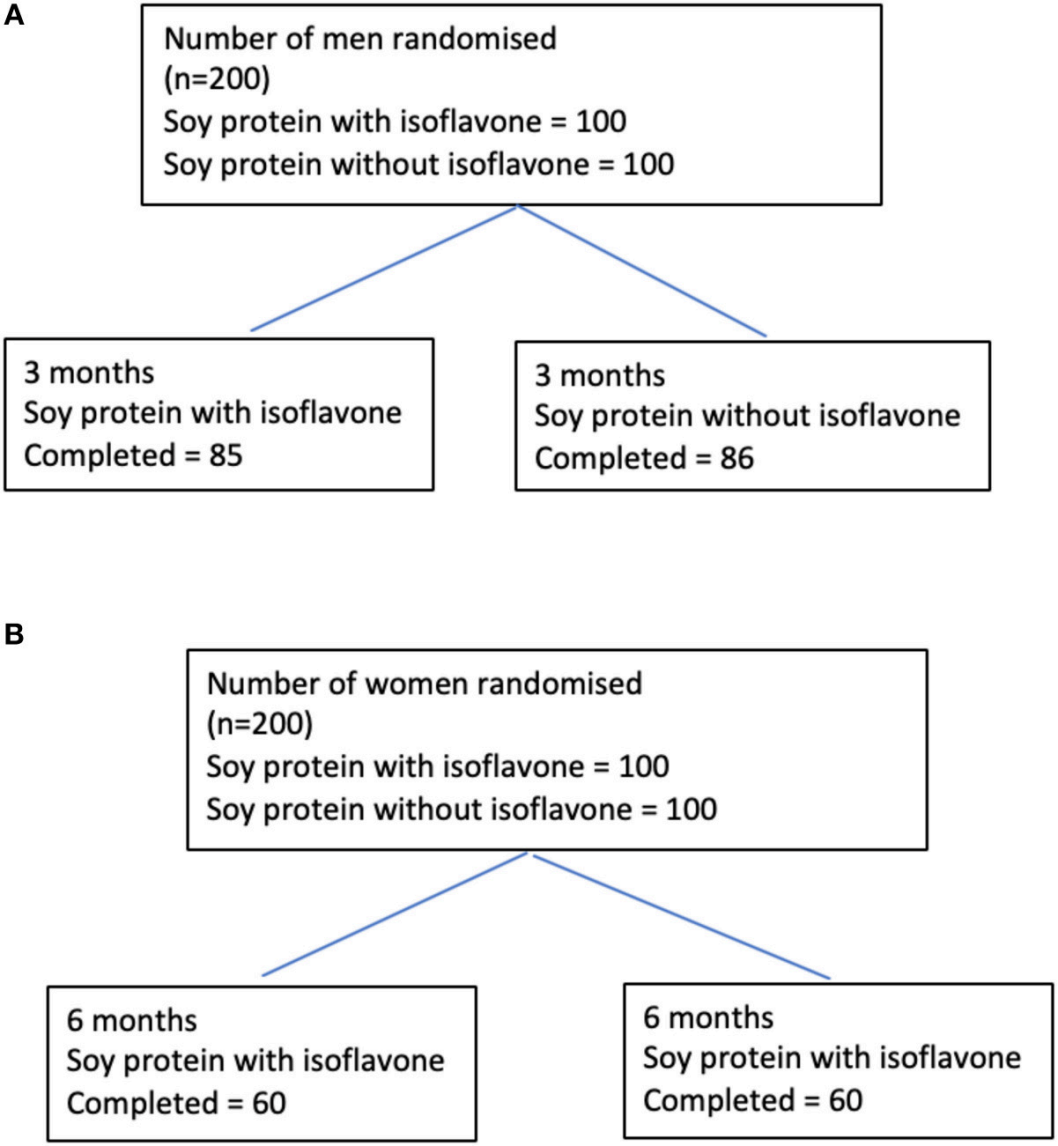
Flow diagrams detailing the participants through studies 1 and 2 from recruitment to the end of the studies. **(A)** Flow diagram of participants through the study 1. **(B)** Flow diagram of participants through the study 2.

Study 1 involved 200 men aged between 45 and 75 years with T2DM, low early morning total testosterone concentrations (total testosterone less than the lower level of the reference range of 12 nmol/L) and normal gonadotropins who participated in a randomized double blind parallel study investigating the effect of soy isoflavones on serum testosterone concentrations (17). They were randomized either to intake of 15 g soy protein with 66 mg of isoflavones (SPI) per day or 15 g soy protein alone without any isoflavones (SP) per day for 3 months in the form of snack bars (Figure 1A). Study 2 involved 200 healthy women within 2 years of the onset of their menopause who were recruited (20) to assess the impact of high dose soy isoflavones on bone turnover markers. They were randomized into either the SPI group (15 g soy protein with 66 mg of isoflavones) or SP group (15 g soy protein alone, isoflavone free) daily intake for a period of 6 months in the form of snack bars (Figure 1B). Thyroid function tests including fT3, fT4, and TSH were secondary outcomes for both studies.

Participants were required to avoid food products containing soy, alcohol, mineral, or vitamin supplementation. All patients were euthyroid and none of the patients were on thyroxine supplementation or on medications, which are known to affect thyroid function. The snack bars were consumed twice daily between meals. Dietary reinforcement together with measurement of plasma isoflavone concentrations was undertaken at each visit to ensure adherence. All subjects gave their written informed consent. Both studies received ethical approval by the Research Ethics Committee (East Yorkshire & North Lincolnshire Research Ethics Committee, ref: 09/H1304/45 and 09/1304/45). Computer-generated block randomization list was performed by Essential Nutrition Ltd., Brough, UK, who held the randomization codes for both the studies.

TSH, fT_3_, and fT_4_ assays were performed on an Abbott Architect i4000 immunoassay analyser (Abbott Diagnostics Division, UK) as batched samples. Analytical performance in terms of imprecision at discreet levels (% CV, Mean) for fT_4_ was 4%, 13.7 pmol/L, for fT3 was 11.2%, 0.33 nmol/L, and that of TSH was 4.2%, 1.32 mU/L. The rT_3_ concentrations were measured in duplicate by competitive radioimmunoassay (total rT3 RIA, lot R-EW-125-1614A; Radim Deutschland GmbH, Freiburg, Germany) according to the manufacturer's instructions as a batched analysis. The assay range was 0.04–3.10 nmol/L and inter-assay %CV was <11.2%. The isoflavones were extracted and analyzed from serum by LGC, Fordham, Cambridgeshire, UK using isotope-dilution LC-MS/MS (21). LC-MS/MS was conducted using a Sciex4000Qtrap with separation achieved using a C18 column and mobile phases of acetonitrile and water, both containing acetic acid (17).

### Study product

“The intervention consisted of a snack bar containing 7.5 g isolated soy protein powder (Solcon F, Solbar Industries, Israel) with 33 mg of isoflavones (SPI) (Solgen 40, Solbar Industries, Israel) given twice daily between meals (15 g soy protein and 66 mg of isoflavones per day), or 7.5 g of the isolated soy protein given twice daily (15 g soy protein per day without isoflavones per day) as control (SP). The latter had an isoflavone concentration of <300 parts per billion following serial alcohol extraction by Dishman Ltd., India (20); and product isoflavones assayed by FERA, Sand Hutton, UK (20). Analysis showed the composition of the dose materials to be 12% glycitein, 35% daidzein, and 54% genistein as aglycones. 90% of isoflavones were in the primary glucoside form, with the remaining 10% as aglycones as malonyl and acetyl glucosides. The snack bars were consumed twice daily between meals for 6 months. The study bars were prepared and packaged by Halo foods, Swindon, UK.” Soy bars were identical and had similar macronutrient content and a tasting panel had determined that there was no discernible difference between the 2 products.

### Statistical analysis

This is a *post-hoc* exploratory analysis of two studies to understand the impact of high dose isoflavones on rT_3_ concentrations and their correlation with other serum thyroid hormone measurement parameters tests including fT_3_, fT_4_, and TSH hence apriori power calculation was not done for changes in rT3.

Baseline continuously distributed data is presented as median (25/75th centiles); categorical data by *n* (%). Within-group differences (difference between 12 weeks/24 weeks values and baseline values) are shown for each treatment group separately by a mean and a standard deviation (SD). Between-group comparisons were performed using the independent sample *t*-test. The *t*-test assumes equal variance between groups. Changes of rT3 concentrations (ΔrT3) with each supplementation was correlated with changes in fT3 (ΔfT3), changes in fT4 (ΔfT4) and changes in TSH (ΔTSH) by Spearman correlation coefficient method. For all statistical analyses, a two-tailed *P* < 0.05 was considered to indicate statistical significance. Statistical analysis was performed using the STATA statistical computer package (StataCorp 2013. *Stata Statistical Software:* StataCorp LP, USA).

## Results

In Study 1, there was a significant increase in rT_3_ with SPI supplementation [0.41 (0.12) vs. 0.45 (0.14) nmol/L] at 3 months compared to SP supplementation [0.43 (0.10) vs. 0.40 (0.15) *p-*value < 0.001] (Tables 1, 2) in men with type 2 diabetes. SPI supplementation increased TSH within 3 months [Mean (SD)] [1.81 (0.92) vs. 3.23 (1.03) mU/L] compared to SP supplementation [1.82 (0.93) vs. 1.96 (1.11) mU/L]. Conversely, SPI supplementation decreased free T_4_ within 3 months [12.68 (1.90) vs. 11.09 (2.00) pmol/L] compared to SP supplementation [13.06 (1.74) vs. 12.74 (1.62) pmol/L]. There were no changes in fT_3_ with 3 months of either SPI or SP supplementation. There was a significant correlation between changes in rT_3_ with TSH (*r* = 0.52; *p* = 0.03) but no correlation with changes in fT_3_ (*r* = 18; *p* = 0.81) and fT_4_ (*r* = 13; *p* = 0.62 0.81).

**Table 1:** Changes in reverse T3 at 3 months for study 1 and at 3 and 6 months for study 2.

**Changes in reverse T3 (rT3)**						
	**Baseline**	**3 months**	***p-*****value (baseline vs. 3 months between groups)**	**6 months**	***p-*****value (baseline vs. 6 months between groups)**	***p-*****value (3 vs. 6 months between groups)**
**STUDY 1**						
SPI	0.41 (0.12)	0.45 (0.14)	< 0.001
SP	0.43 (0.10)	0.40 (0.15)	
**STUDY 2**						
SPI	0.33 (0.12)	0.37 (0.09)	< 0.001	0.31 (0.13)	0.81	0.001
SP	0.33 (0.11)	0.33 (0.12)		0.30 (0.12)	

*SPI, 15 g soy protein with 66mg of isoflavones; SP, 15 g soy protein alone isoflavone free. Results in Mean (SD)*.

**Table 2:** Baseline characteristics of study 1 participants.

**Parameters**	**Soy protein without isoflavone**	**Soy protein with isoflavone**
Body mass index (kg/m^2^)	31.6 (29.2, 35.0)	31.8 (28.8, 34.7)
Age (years)	52.0 (50.0, 55.0)	52.0 (50.0, 55.0)
HbA1c (mmol/mol)	58 (53, 64)	56 (52, 60)
Duration of diabetes (years)	7.9 (4.4, 9.1)	7.3 (4.2, 8.8)
HbA1c (mmol/mol)	58 (53, 64)	56 (52, 60)
fT4 (pmol/L)	13.0 (12.0, 14.0)	12.0 (12.0, 14.0)
fT3 (pmol/L)	4.6 (4.2, 4.9)	4.6 (4.2, 5.0)
TSH (mU/L)	1.6 (1.2, 2.5)	1.6 (1.2, 2.4)

*Data are provided as medians (25/75th centiles). fT4, free thyroxine; fT3, free tri-iodo thyronine; TSH, thyroid stimulating hormone*.

In Study 2 in healthy women within 2 years of menopause (Table 3), SPI supplementation increased rT_3_ [0.33 (0.12) vs. 0.37 (0.09) nmol/L] compared to SP supplementation (*p* < 0.001) within 3 months. The rT_3_ decreased after 6 months 0.31 (0.13) of SPI supplementation and was comparable to baseline (*p*-value = 0.81). Mean TSH increased significantly [mean (SD) 1.58 (0.93) vs. 2.61 (1.24) mU/L, *p* < 0.01] with a corresponding reduction in mean fT_4_ [13.5 (2.2) vs. 11.2 (1.8) pmol/L, *p* < 0.01] from baseline to 3 months. There was a significant correlation between changes in rT_3_ with TSH (*r* = 0.612; *p* = 0.02) but no correlation with changes in fT_3_ (*r* = 22; *p-*value = 0.42) or fT_4_ (*r* = 18; *p-*value = 0.38) at 3 months. There was no correlation between changes in rT_3_ with changes in TSH, fT_4_ and fT_3_ at 6 months.

**Table 3:** Baseline characteristics of study 2 participants.

**Parameters**	**Soy protein without isoflavone**	**Soy protein with isoflavone**
Body mass index (kg/m^2^)	24.6 (22.7, 28.4)	26.3 (24.3, 30.7)
Age (years)	52 (50, 55)	52 (49, 56)
fT4 (pmol/L)	13 (12, 14)	13 (13, 15)
fT3 (pmol/L)	4.7 (4.3, 4.9)	4.6 (4.3, 5.1)
TSH (mU/L)	1.6 (0.9, 2.3)	1.5 (0.9, 2.2)

*Data are provided as medians (25/75th centiles). fT4, free thyroxine; fT3, free tri-iodo thyronine; TSH, thyroid stimulating hormone*.

There were no changes in TSH and fT_4_ between 3 and 6 months. There were no differences in the fT_3_ between both preparations.

## Discussion

There was a significant increase in rT_3_ in both studies after 3 months of high dose isoflavone supplementation, whereas the rT_3_ values did not differ in isoflavone-free soy, suggesting that it is the isoflavone component that is responsible for the rT_3_ changes seen. However, in the study 2 involving post-menopausal women the rT_3_ decreased to baseline values after 6 months of SPI supplementation, suggesting that the isoflavone induced changes are transient and return to normal pretreatment thyroid homeostasis over a 6-months period.

In both studies, there was a reduction in fT4 and a corresponding rise in TSH, suggesting that the feedback response of the hypothalamo-pituitary-thyroid axis was intact. In situations where the thyroid cannot maintain thyroid hormone production, either due to an autoimmune process and/or lack of the essential trace element iodine, there would be a shift in thyroidal production from a 20:1 T4:T3 ratio to a ratio more in favor of T3. A preferential production and secretion of T3 compared to T4 could potentially explain why there were no significant changes in fT3 despite changes in TSH and fT4. Dose and duration of SPI consumption might not yet be sufficient to also decrease serum fT3 considering that the majority of T3 is generated outside of the thyroid gland by Type 1 and Type II 5′-deiodinase activities. Furthermore, SPI isoflavones might enhance (hepatic and/or gastrointestinal) T4 elimination by hepatic enzyme induction, as frequently observed for drugs (e.g., phenobarbital) and xenobiotics, while serum T3 concentration are still maintained (22–24). However, given the uniform response of the increase in rT3 in response to high dose isoflavones, this would suggest that this change is not idiosyncratic. Interestingly, changes in serum rT3 in the SPI isoflavone consuming groups were similar in T2DM male patients and post-menopausal women, which in our opinion would exclude effects solely restricted to T2DM patients or post-menopausal women.

Serum T4 is metabolized either to the active thyroid hormone T3 or to the inactive rT3 in a reciprocal manner depending upon the relative actions of the tissue deiodinase enzymes types 1 to 3. The mechanism of the increase in rT3 seen in both studies is unclear. One of the main sources of rT3 is the peripheral conversion of thyroxine to rT3. The enzyme responsible for this is deiodinase type 3 and it could be hypothesized that isoflavones may activate deiodinase type 3 (25), but no data has been reported on direct stimulation of expression of any of the deiodinases by isoflavones. Degradation of rT3 is mainly accomplished by hepatic and renal deiodinase type 1, but also by deiodinase type 2 (25). Therefore, another plausible explanation would be that isoflavones (transiently) inhibit hepatic deiodinase 1 (and/or extrahepatic deiodinase 2) (25), which would lead to accumulation of rT3 in blood. However, in rat-studies we have shown that deiodinase type 1 activity in the liver is increased after 16 weeks of isoflavone treatment (15), which might be due to higher hepatic uptake and exposure to thyroid hormone which may be displaced by flavonoids from its binding to transthyretin (16, 26). Administration of high concentrations of rT3, administered in rodent experiments, did not affect serum TSH and thyroid hormones, albeit type 2 deiodinase activity was inhibited and hepatic gene expression was affected (27) The short half-life and rapid turnover of rT3 might result in these transient changes and flavonoid effects on deiodinase isoenzymes and transthyretin might mainly manifest as variations in rT3 serum concentrations which is only weakly bound to serum distribution proteins compared to T4 and T3. Whether the thyroid itself is also affected by isoflavones and/or responds to altered TSH concentration, which might induce activity and expression of type 1 and type 2 deiodinases and thus maintain T3 concentration, requires detailed kinetic studies (28). There may be multiple mechanisms through which isoflavones act on thyroid hormone metabolism, distribution and/or transport systems with a complexity that needs further elucidation. It is also unknown if the isoflavone effects would be different between patients with and without autoimmune thyroid disease.

Reduced circulating levels of fT_3_ are a sensitive marker of ill health especially in context of elevated rT3 (29). In a population of elderly men, who were independently living, serum rT_3_ concentrations increased with age and the presence of comorbidities (29). Higher serum rT_3_ concentrations may result from decreased peripheral metabolism of TH due to the aging process itself and/or disease and may reflect a catabolic state (29). Indeed, fasting, cold exposure, and even minor infective or inflammatory disease are sufficient to reduce serum fT_3_ and elevate rT_3_ concentrations in otherwise-healthy individuals (30, 31).

In a cohort of elderly people, baseline serum rT_3_ concentration was associated with all-cause mortality during a 9-years period of follow up, suggesting that rT_3_ may be a more sensitive marker for non-thyroidal illness than fT_3_ (32). No significant mortality associations were found with serum fT_3_, fT_4_ or TSH concentrations. However, in men lower serum TSH, and in women higher rT_3_ concentrations, predicted disability (32). These findings broadly confirm those of previous studied cohorts of older people (33, 34), and are in line with the known pathophysiological mechanisms whereby low fT_3_ and higher rT_3_ concentrations are non-specifically associated with increasing ill health and disease burden (31). In the elderly, high rT_3_ is a stronger predictor of all-cause mortality than low fT_3_ independent of disease burden (32).

Dietary intake of isoflavones in Asian soy diets has been estimated to be in the range of 30–50 mg per day of combined isoflavone aglycone equivalents (35, 36). In Western countries an average daily intake of ~2 mg isoflavones is seen though estimated to be 16 mg in vegetarians (37); therefore, the dose of 66 mg of isoflavones used in this study may be considered to be in the pharmacological range for this study purpose.

In conclusion, these studies show that the isoflavone component of soy protein transiently increases rT_3_ concentrations when supplemented over a period of 3 months but afterwards rT_3_ concentrations reverted to baseline at 6 months, perhaps due to deiodinase 3 inhibition. These changes were mirrored in the TSH values, suggesting that high dose isoflavones may transiently impair thyroid homeostasis, though it is not clear if this would impact clinically on general health.

## Author contributions

TS, EK, and SA devised the study. JK and ER measured the rT3 whilst. AR and SD performed the statistical analysis. All authors contributed to the writing and final review of the manuscript.

### Conflict of interest statement

The authors declare that the research was conducted in the absence of any commercial or financial relationships that could be construed as a potential conflict of interest.
